# Antimicrobial prescribing in a secondary care setting during the COVID-19 pandemic

**DOI:** 10.1093/jacamr/dlad117

**Published:** 2023-11-13

**Authors:** Michael M Tadros, Marian S Boshra, Michael Scott, Glenda Fleming, Fidelma Magee, Mohammad I Hamed, Ahmed Abuelhana, Aaron Courtenay, Heba F Salem, Kathryn Burnett

**Affiliations:** School of Pharmacy and Pharmaceutical Sciences, Ulster University, Coleraine, UK; Clinical Pharmacy Department, Faculty of Pharmacy, Misr University for Science and Technology (MUST University), P.O. Box 12566, Giza, Egypt; Clinical Pharmacy Department, Faculty of Pharmacy, Beni-Suef University, P.O. Box 62514, Beni-Suef, Egypt; Medicines Optimisation and Innovation Centre (MOIC), Antrim Area Hospital, Antrim, UK; Medicines Optimisation and Innovation Centre (MOIC), Antrim Area Hospital, Antrim, UK; Pharmacy Department, Northern Health and Social Care Trust (NHSCT), Antrim, UK; Clinical Pharmacy Department, Faculty of Pharmacy, Misr University for Science and Technology (MUST University), P.O. Box 12566, Giza, Egypt; School of Pharmacy and Pharmaceutical Sciences, Ulster University, Coleraine, UK; School of Pharmacy and Pharmaceutical Sciences, Ulster University, Coleraine, UK; Department of Pharmaceutics and Industrial Pharmacy, Faculty of Pharmacy, Beni-Suef University, P.O. Box 62514, Beni-Suef, Egypt; Regional Pharmaceutical Procurement Service, Northern Health and Social Care Trust (NHSCT), Antrim, UK

## Abstract

**Background:**

Increased antimicrobial resistance patterns lead to limited options for antimicrobial agents, affecting patient health and increasing hospital costs.

**Objectives:**

To investigate the antimicrobial prescribing patterns at two district hospitals in Northern Ireland before and during the COVID-19 pandemic.

**Methods:**

A mixed prospective-retrospective study was designed to compare pre- and during pandemic antimicrobial prescribing data in both hospitals using a Global Point Prevalence Survey.

**Results:**

Of the 591 patients surveyed in both hospitals, 43.8% were treated with 402 antimicrobials. A total of 82.8% of antimicrobial prescriptions were for empirical treatment. No significant difference existed in numbers of patients treated or antimicrobials used before and during the pandemic. There was a slight decrease of 3.3% in the compliance rate with hospital antimicrobial guidelines during the pandemic when compared with the pre-pandemic year of 2019, when it was 69.5%. Treatment based on patients’ biomarker data also slightly decreased from 83.5% pre-pandemic (2019) to 81.5% during the pandemic (2021).

**Conclusions:**

There was no overall significant impact of the pandemic on the antimicrobial prescribing patterns in either hospital when compared with the pre-pandemic findings. The antimicrobial stewardship programmes would appear to have played an important role in controlling antimicrobial consumption during the pandemic.

## Introduction

Antimicrobial resistance is an increasing problem worldwide, often resulting from suboptimal patterns of antimicrobial prescribing.^1^ Increased antimicrobial resistance leads to limited options of effective antimicrobial agents, affecting patient health and increasing hospital costs.^2^ Effective optimization of antimicrobial use and optimal dosing regimens of antimicrobials slow the rate of development of antimicrobial resistance. Rates of morbidity and mortality and hospital costs are decreased with prudent use of antimicrobials through antimicrobial stewardship programmes.^2,3^ Antimicrobial resistance before the COVID-19 pandemic was one of the big issues for global public health, but during COVID-19 the priorities changed to concentrate on pandemic management and vaccination.^4^ Several studies in different countries have reported an increased rate of antimicrobial resistance and different antimicrobial prescribing patterns during the COVID-19 pandemic.^5–8^ During the pandemic there was a high rate of patients admitted to hospitals suffering from severe pneumonia and other lung-associated problems, and this led to increased empirical use of antimicrobials, which could be a potential cause of increasing antimicrobial resistance.^9^

The Global Point Prevalence Survey (G-PPS) is a standardized method with a web-based tool used worldwide for monitoring and managing antimicrobial prescribing and resistance patterns for improving patient outcomes and cost-effective antimicrobial therapy, and, as such, was used in this study.^10^

The overall aim of the study was to analyse the antimicrobial prescribing patterns before and during the COVID-19 pandemic within the Northern Health & Social Care Trust (NHSCT) using the G-PPS.^10^ This was to determine the impact of the pandemic (if any) and to assess and compare the quantity and quality of antimicrobial prescribing patterns within the Trust. A secondary aim was to use this information to inform appropriate hospital interventions aiming to promote the prudent use of antimicrobials.

## Methods

### Study design

This study was conducted in Antrim Area and Causeway Hospitals, NHSCT, Northern Ireland, UK. It was designed as a mixed retrospective and prospective based observational study to quantitatively identify, measure and analyse the scope, quantity and quality of antimicrobial prescribing within a secondary care setting, both before and during the COVID-19 pandemic. Data were collected using the standardized G-PPS data collection tool for 2021 within the NHSCT.^11^

### Study setting

The NHSCT, the largest geographical Trust in Northern Ireland, includes Antrim Area Hospital and Causeway Hospital in addition to two other acute hospitals (Mid-Ulster Hospital and Whiteabbey Hospital). This Trust provides health and social care services to a population of approximately 479 000 inhabitants across a geographical area of 1733 square miles.^12^ Antrim Hospital, the largest hospital within the NHSCT in Northern Ireland, is a 503-bed hospital (during the time of the study) serving almost 450 000 people. Causeway Hospital is a 224-bed hospital (during the time of the study). Both hospitals participated before in the G-PPS; they included 36 wards (surgical, medical and mixed wards) with an average of 20 beds on each ward.^12^

### Data collection for the G-PPS survey

In addition to the retrospective (G-PPS) data that had been collected from previous years, data were also collected between May and August 2021 (during the pandemic) using a G-PPS standardized data collection sheet (Table S1, available as Supplementary data at *JAC-AMR* Online), for all inpatients prescribed antimicrobial agents by 8 am on the day of survey for each ward.^11^ G-PPS is a completely anonymous survey for local antimicrobial prescribing practice. For collection the survey required information from paper-based and electronic patient records, including medical notes, nursing notes and the patient’s medication charts, and this was made available after being anonymized by the ward clinical pharmacists (no patient identifiers were available to the investigators while collecting these data).^13^ The investigators had no access to other sources such as a laboratory computer system for patient information. Data collection for this PPS was carried out in accordance with the standardized G-PPS protocol using the G-PPS 2021 data collection forms.^14^ All inpatient wards within Antrim Area Hospital and Causeway Hospital were included in data collection.

### Inclusion and exclusion criteria for the G-PPS

These were based on the standardized methods outlined in the G-PPS protocol, as shown in Table 1.^14^

**Table 1.** Inclusion and exclusion criteria for the Global-PPS 2021^14^

Inclusion criteria	Exclusion criteria
All inpatients admitted on a ward at 8 am on the day of survey All inpatients ‘on antimicrobial agents’ at 8 am on the day of survey Patients receiving an antimicrobial, e.g. every 48 h but receiving this antimicrobial on the survey day Patients receiving surgical prophylaxis, checked in the previous 24 h; patients receiving surgical prophylaxis before 8 am on the survey day	Day hospitalizations and outpatients (ambulatory care patients) Patients admitted after 8 am on the survey day All inpatients ‘on antimicrobial agents’ at 8 am on the day of survey Patients prescribed an antimicrobial in the afternoon on the day of the survey Patients receiving surgical prophylaxis after 8 am on the day of the survey

### Statistical analysis

The statistics and data analysis were conducted using Microsoft Excel^®^ and SPSS^®^ Statistics, Version 27; descriptive analysis such as frequencies and percentages, along with statistical analysis for significance, such as Pearson’s chi-squared test, were employed.

### Data confidentiality and protection

Following the standardized G-PPS 2021 protocol,^14^ all data were completely anonymized. In the G-PPS tool every patient record received a unique non-identifiable survey number. This number was automatically generated by the computer program based on various internal codes. This number identifies the patient uniquely in the G-PPS database. No patient or personal identifiers were recorded. The collected data were used and stored securely, ensuring the confidentiality of all data contained therein. The data collected in this survey were summarized and presented as results of the PPS with no patient or practitioner identifiers ensuring that no sensitive or identifying data were published.

### Ethical approval

Ethical approval was granted for this study by Ulster University Biomedical Sciences Ethics filter committee (FCBMS-21-015), and approved by the Research Governance Department of the NHSCT before commencing. The study was also approved by the Research and Ethics Committee (REC) of the Faculty of Pharmacy, Beni-Suef University (REC-H-PhBSU-22017).

## Results

### Characteristics of participating hospitals and surveyed patients during the pandemic over May–August 2021

**Figure 1.**
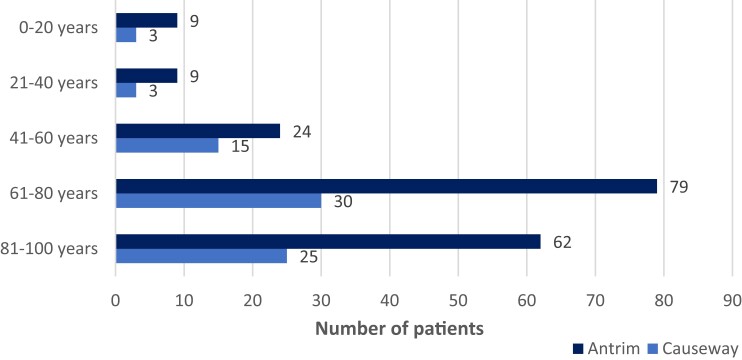
Age of patients treated with antimicrobial therapy within Antrim Area and Causeway Hospitals during the pandemic over May–August 2021.

The total number of patients surveyed in both hospitals was 591, with 414 (70.1%) from Antrim Area Hospital and 177 (29.9%) from Causeway Hospital. There was a total of 36 inpatient wards included in the survey—27 medical, 3 surgical, 2 ICUs and 4 wards of mixed activities (i.e. identified by major activity level, e.g. surgical ward or ICU ward taking overflow from medicine). The bed occupancy within Antrim Hospital was 86.4%, 414 out of 479 available beds were occupied. The bed occupancy in Causeway Hospital was 81.9%, 177 out of 216 available beds were occupied. Total bed occupancy for both hospitals during the pandemic over May–August 2021 was 85%: 591 out of the 695 available beds were occupied. Based on the ward activity in both hospitals the bed occupancy was 86%, 80.8% and 70.5% for medical, surgical and ICU wards, respectively. Of the 259 (43.8%) patients on antimicrobial therapy, 183 (70.7%) were inpatients in Antrim Hospital and 76 (29.3%) were inpatients in Causeway Hospital. The average percentage of patients being prescribed antimicrobials was 41.1%, 59.2% and 58.3% on medical, surgical and ICU wards, respectively. The majority of patients being prescribed antimicrobial therapy during the pandemic over May–August 2021 were over 60 years of age (*n* = 196, 75.6%) as shown in Figure 1. The average age was 69.2 years (95% CI: 66.75–71.73) and the median was 75 years.

### Antimicrobial prescription patterns during the pandemic over May–August 2021

**Figure 2.**
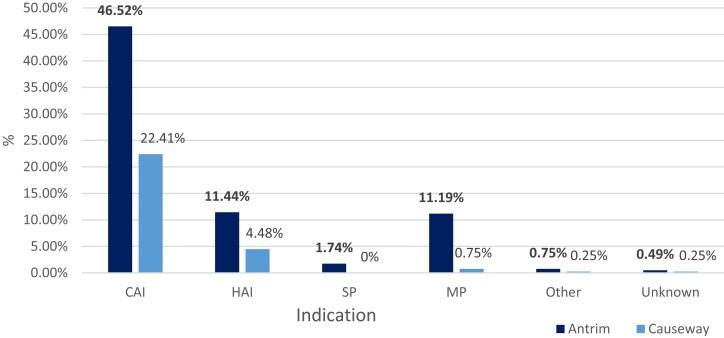
Percentage of indications for antimicrobial prescriptions within Antrim Area and Causeway Hospitals during the COVID-19 pandemic over May–August 2021. CAI, community-acquired infection; HAI, hospital-acquired infection; MP, medical prophylaxis; SP, surgical prophylaxis.

A total of 402 antimicrobials were prescribed: 290 (72.1%) to patients in Antrim Hospital and 112 (27.9%) to patients in Causeway Hospital. Parenteral antimicrobials (*n* = 226, 56.2%) were prescribed more often than oral antimicrobials (*n* = 176, 43.8%); however, there was no significant difference (*P* < 0.05; Pearson’s chi-squared test) in parenteral and oral antimicrobial use between both hospitals during the pandemic, whereas before the pandemic in 2019, 56.6% parenteral antimicrobials and 43.4% oral antimicrobials were prescribed. As shown in Figure 2, indications for the 402 antimicrobial prescriptions during the pandemic were (according to the type of indication per the standardized G-PPS 2021): community-acquired infections (CAIs) (*n* = 276, 68.7%; compared with 59.7% before the pandemic), hospital-acquired infections (HAIs) (*n* = 64, 15.92%), surgical prophylaxis (*n* = 7, 1.74%) and medical prophylaxis (MP) (*n* = 48, 11.9%); the indication was unknown or other for 7 (1.74%) of antimicrobial prescriptions. The dominant infections for all inpatients being prescribed antimicrobials during the pandemic were pneumonia or lower respiratory tract infections (LRTIs) (*n* = 101, 25.1%; compared with 37.5%, *n* = 145 before the pandemic in 2019); intra-abdominal sepsis (*n* = 72, 18%); and upper urinary tract infections (*n* = 42, 10.4%). Prescriptions were also found for general medical prophylaxis without a specific diagnostic site (*n* = 35, 8.7%). Of the 259 inpatients prescribed an antimicrobial, 136 (52.5%) were male and 123 (47.5%) were female. Of the surveyed patients on antimicrobial therapy during the pandemic (*n* = 259), 156 (60.2%) were prescribed one antimicrobial and 103 (39.8%) were prescribed two or more antimicrobials; pre-pandemic (2019 PPS) 248 patients were prescribed antimicrobials, with 57.3% prescribed one antimicrobial and 42.7% prescribed two or more antimicrobials.

### Antimicrobial choice during the pandemic over May–August 2021

**Figure 3.**
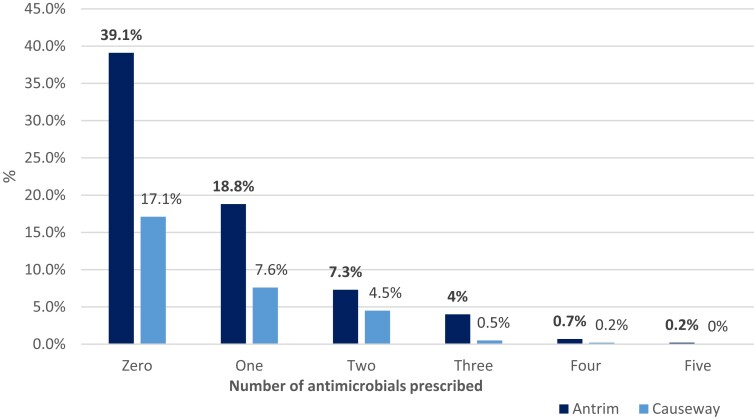
Percentage of antimicrobials prescribed per patient surveyed in Antrim Area and Causeway hospitals during the pandemic over May–August 2021.

A total of 103 (39.8%) patients prescribed antimicrobials were treated with more than one antimicrobial on the day of the G-PPS survey (Figure 3). Within the two hospitals, the most frequently prescribed antimicrobial groups were combinations of penicillins, including β-lactamase inhibitors (J01CR), penicillins with extended-spectrum (J01CA) and imidazole derivatives (J01XD), which accounted for 24.4%, 13.4% and 9.7% of the total antimicrobial prescriptions, respectively; before the pandemic in 2019 the proportions were 24% for J01CA and 17.8% for J01CR. The most frequently prescribed antimicrobial agents during the pandemic were piperacillin/enzyme inhibitor (*n* = 66, 16.4%), amoxicillin (*n* = 48, 12%), metronidazole (*n* = 39, 9.7%), co-amoxiclav (*n* = 33, 8.2%) and clarithromycin (*n* = 26, 6.5%), which were relatively similar to before the pandemic—the most frequently prescribed antimicrobials in 2019 before the pandemic were amoxicillin (*n* = 67, 17.3%), piperacillin/tazobactam (*n* = 61, 15.8%), clarithromycin (*n* = 36, 9.3%), co-amoxiclav (*n* = 33, 8.5%) and metronidazole (*n* = 25, 6.5%).

### Antimicrobial quality indicators

Treatment based on biomarker data such as C-reactive protein (CRP) or WBC count refers to whether or not biomarker results were used to initiate antimicrobial treatment. That approach is helpful in reducing the initiation and unnecessary duration of the antimicrobial treatment.^14^ Antimicrobial therapy was initiated based on biomarker data for 211 (81.5%) out of the 259 patients treated with antimicrobials. Treatment based on biomarker data for patients at Causeway Hospital (*n* = 69, 90.8%) was higher than at Antrim Area Hospital (*n* = 142, 77.6%). The two hospitals utilized the two biomarkers, CRP and WBC count, as noted in patients’ medical records. CRP directed 171 (81%) patients’ therapy whereas WBC count guided 40 (19%). Laboratory culture requests were made for 141 (54.4%) of the 259 patients: 123 (67.2%) from Antrim and 18 (23.7%) from Causeway Hospital.

Indication therapy was documented in patients’ medical files for 382 (95%) of the antimicrobial prescriptions. The recording of a review or stop date for the antimicrobial was documented more often within Causeway Hospital (58%) than Antrim Area Hospital patients (40.7%) (Table 2). Empirical treatment (prescribing an antimicrobial according to local guidelines or when microbiological results were unavailable on the surveyed day of the PPS),^10^ was identified in 333 (82.8%) of the 402 prescriptions. Only 69 (17.2%) antimicrobials were being prescribed as a targeted therapy, when the microbiological results directed the therapy on the survey day.

**Table 2.** Documentation of indication for antimicrobial treatment and date to stop or review antimicrobial therapy within patients’ medical notes in Antrim Area and Causeway hospitals (*n* = number of antimicrobial prescriptions) during the pandemic over May-August 2021

Documentation	Antrim Area Hospital, *n* (%)	Causeway Hospital, *n* (%)	Total, *n* (%)
Indication for treatment recorded			
Yes	285 (98.3%)	97 (86.6%)	382 (95%)
No	5 (1.7%)	15 (13.4%)	20 (5%)
Stop or review date documented			
Yes	118 (40.7%)	65 (58%)	183 (45.5%)
No	172 (59.3%)	47 (42%)	219 (54.5%)

**Figure 4.**
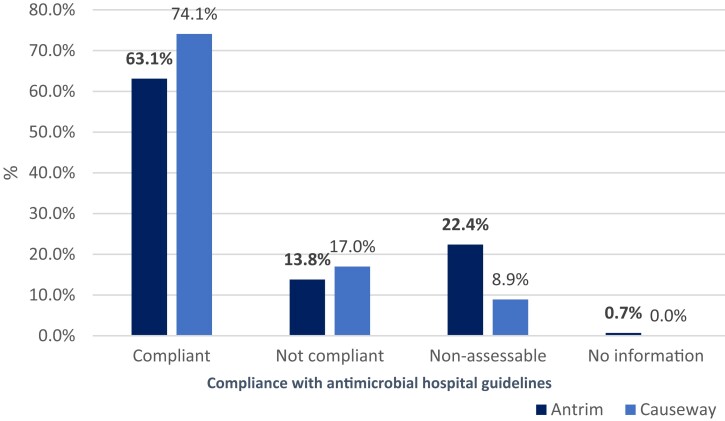
Compliance of antimicrobial prescriptions with local hospital antimicrobial guidelines within Antrim Area Hospital and Causeway Hospital during the pandemic over May–August 2021. Compliant: when the antimicrobial choice is in compliance with local guidelines or infection specialist advice. Not compliant: not compliant with local policy or infection specialist advice. Non-assessable: no local guidelines for the specific indication. No information: because the indication is unknown.^14^

It was found that 266 (66.2%) antimicrobial prescriptions were compliant with local hospital guidelines, 59 (14.7%) were non-compliant, 75 (18.6%) were non-assessable due to the absence of local guidelines for the specific indication, and 2 (0.5%) had no information because of unknown diagnosis/indication. As shown in Figure 4, the percentage of antimicrobial therapy non-compliant with hospital guidelines in Causeway Hospital (*n* = 19, 17%) was higher than in Antrim Hospital (*n* = 40, 13.8%). There was a significant difference (*P *= 0.015; Pearson’s chi-squared test) in compliance with hospital antimicrobial guidelines (Figure 4) between the hospitals, with compliance being higher in Causeway Hospital than Antrim Hospital.

### Evaluation of antimicrobial prescribing patterns

Linking the current data to a retrospective analysis of the records within the Trust and over six timepoints between 2009 and 2021 using the G-PPS tool, a total of 5404 patients were surveyed during their inpatient stay: 1203 in 2009, 876 in 2011, 1526 in 2015, 600 in 2017, 608 in 2019 and 591 in 2021. The overall features of antimicrobial prescribing patterns across the six timepoints are shown in Table 3.

**Table 3.** General characteristics and antimicrobial prescription patterns of patients surveyed over six timepoints (2009, 2011, 2015, 2017, 2019 and 2021)^10^

	2009, *n* (%)	2011, *n* (%)	2015, *n* (%)	2017, *n* (%)	2019, *n* (%)	
Characteristics						2021, *n* (%)
Number of hospitalized patients	1203	876	1526	600	608	591
Number of treated patients (% of total inpatients)	374 (31.1)	298 (34.0)	567 (37.2)	269 (44.8)	248 (40.8)	259 (43.8)
Median age of treated patients, years	70	71	72	72	72	75
Gender (% of treated patients)						
Male	182 (48.6)	161 (54.0)	270 (47.6)	125 (46.5)	111 (44.8)	136 (52.5)
Female	192 (51.3)	137 (46.0)	297 (52.4)	144 (53.5)	137 (55.2)	123 (47.5)
Number of prescribed antimicrobials	531	403	818	408	387	402
Number of antimicrobials prescribed per patient surveyed	1.42	1.35	1.44	1.51	1.56	1.55
Route of administration (% of antimicrobial prescriptions)						
Oral	200 (37.7)	184 (45.7)	257 (31.4)	155 (37.9)	168 (43.4)	176 (43.8)
Parenteral	331 (62.3)	219 (54.3)	561 (68.6)	252 (61.6)	219 (56.6)	226 (56.2)
Indication (% of antimicrobial prescriptions)						
Community-acquired infection	304 (57.0)	279 (69.2)	554 (67.7)	234 (57.4)	231 (59.7)	276 (68.6)
Hospital-acquired infection	145 (27.2)	76 (18.9)	145 (17.7)	131 (32.1)	120 (31.0)	64 (16)
Surgical prophylaxis	60 (11.3)	29 (7.2)	66 (8.1)	3 (0.7)	1 (0.3)	7 (1.7)
Medical prophylaxis	24 (4.5)	19 (4.7)	33 (4.0)	37 (9.1)	32 (8.3)	48 (12)
Diagnosis site (% of antimicrobial prescriptions)						
Central nervous system	4 (0.8)	6 (1.5)	8 (1.1)	0 (0.0)	2 (0.5)	7 (1.7)
Eye	0 (0.0)	0 (0.0)	0 (0.0)	0 (0.0)	0 (0.0)	0 (0.0)
Otolaryngology	18 (3.4)	14 (3.5)	13 (1.6)	21 (5.1)	12 (3.1)	12 (3)
Respiratory	169 (31.7)	136 (33.7)	286 (35.0)	135 (33.1)	159 (41.1)	126 (31.3)
Cardiovascular	6 (1.1)	1 (0.2)	3 (0.4)	2 (0.5)	3 (0.8)	1 (0.2)
Gastrointestinal tract	95 (17.8)	55 (13.6)	132 (16.1)	10 (2.5)	54 (14.0)	85 (21.1)
Skin, soft tissue, bone and joint	89 (16.7)	76 (18.9)	110 (13.4)	28 (6.9)	27 (7.0)	24 (6)
Urinary tract	69 (12.9)	51 (12.7)	87 (10.6)	57 (14.0)	52 (13.4)	71 (17.7)
Genitourinary and obstetrics	30 (5.6)	22 (5.5)	54 (6.6)	14 (3.4)	5 (1.3)	10 (2.5)
Undefined site	57 (10.7)	42 (10.4)	117 (14.3)	135 (33.1)	67 (17.3)	64 (16)
Neonatal	0 (0.0)	0 (0.0)	8 (1.0)	6 (1.5)	6 (1.6)	2 (0.5)

**Figure 5.**
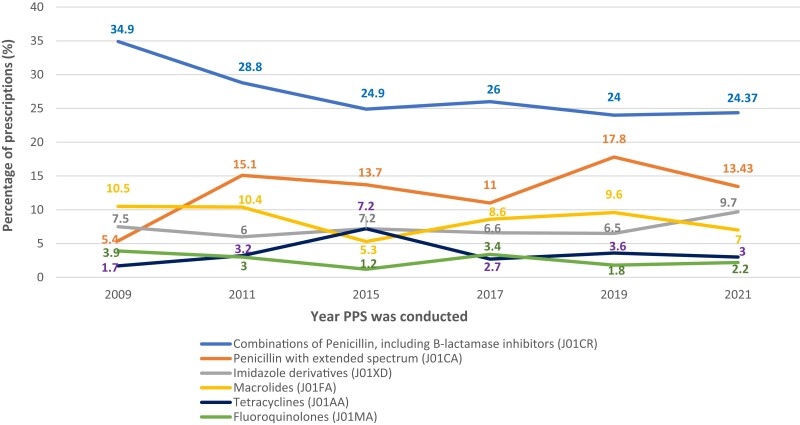
Prescribing patterns of tetracyclines (J01AA), combinations of penicillins, including β-lactamase inhibitors (J01CR), fluoroquinolones (J01MA), penicillins with extended spectrum (J01CR), macrolides (J01FA) and imidazole derivatives (J01XD) in the study hospitals across the six timepoints (2009, 2011, 2015, 2017, 2019 and 2021) (% of prescribed antimicrobials). *Note:* In the Anatomical Therapeutic Chemical (ATC) classification system, the active substances are divided into different groups according to the organ or system on which they act according to their therapeutic, pharmacological and chemical properties. ‘J’ refer to anti-infective for systematic use. The ATC index 2022 is published by the WHO Collaborating Centre for Drug Statistics Methodology.^15^

The majority of antimicrobial agents being frequently prescribed across all timepoints were combinations of penicillins, including β-lactamase inhibitors (J01CR), which decreased from 34.9% in 2009, to 24% in 2019 and 24.4% in 2021. Figure 5 illustrates the overall changes in antimicrobial usage over the time.

The antimicrobial quality indicators for both hospitals across the six timepoints can be seen in Table 4.^10^ Compliance with the local hospital antimicrobial guidelines slightly decreased from 69.5% in 2019 to 66.2% in 2021. Although the documented indication started to increase over time, there was a slight decrease to 95% in 2021 compared with 95.9% in 2019. Therapy initiation based on biomarker data increased significantly over the time from 61.5% in 2015 to 83.5% in 2019, but slightly decreased to 81.5% in 2021 (during the pandemic).

**Table 4.** Quality indicators in antimicrobial prescribing in the study hospitals across six timepoints (2009, 2011, 2015, 2017, 2019 and 2021)^10^

Indicator	2009, *n* (%)	2011,^a^ *n* (%)	2015, *n* (%)	2017, *n* (%)	2019, *n* (%)	2021, *n* (%)
Compliant with hospital	250 (47.1%)	414 (67.0%)	641 (78.4%)	282 (69.0%)	269 (69.5%)	266 (66.2%)
antimicrobial guidelines						
Indication for treatment	471 (88.7%)	542 (87.7%)	741 (90.6%)	378 (92.4%)	371 (95.9%)	382 (95%)
was recorded						
Treatment was based on	NA	NA	503 (61.5%)	NA	207 (83.5%)	211 (81.5%)
biomarker data						

*n*, number of antimicrobial prescriptions; NA, not available.

^a^Includes some data collected for Craigavon Area Hospital Southern Health and Social Crae Trust (SHSCT).

## Discussion

In this study based on the 2021 PPS data collection (during the pandemic), the bed occupancy of the two hospitals (85%) was almost comparable with that previously published before the pandemic by Harvey *et al.*^16^ in 2018 regarding statistics from Northern Ireland Hospital Information. These data showed occupancy of 83.5% in secondary care hospitals in 2018/2019. The 2021 PPS data showed that more males (52.5%) were prescribed antimicrobial therapy than females (47.5%) and the majority of treated inpatients were over 60 years of age (75.6%); comparison with other published PPS studies in Northern Ireland between 2009 and 2015 shows these results to be slightly higher in terms of age and the inverse of gender distribution of treated inpatients.^10^

The patients treated with antimicrobials represented 37.3% of the total 5404 surveyed across all timepoints. This was comparable to previous studies that showed one-third of inpatients being prescribed antimicrobials.^17^ The number of patients prescribed antimicrobials increased over time, recorded as 43.8% in 2021 PPS data (during the pandemic), which was higher than for the pre-pandemic year in 2019 (40.8%).

The numbers of prescribed antimicrobials before and during the pandemic were relatively similar with no significant difference. In 2021, 60.2% of patients were prescribed only one antimicrobial, compared with 57.3% in 2019. This showed that there was no impact of the COVID-19 pandemic on the total number of prescribed antimicrobials for each individual patient before and during the pandemic. These findings contrast with those of Lai *et al*.,^18^ who concluded there was a significant increase in antimicrobial prescribing during the COVID-19 pandemic in secondary care settings in China. The conclusion of Lai *et al.* could be attributed to the different health systems, antimicrobial stewardship programmes, policy and implementation in China and the UK.

An average of 1.55 antimicrobials per individual patient was shown by the 2021 data (during the pandemic), which was nearly equal to the pre-pandemic results (1.56), but slightly greater than the average of other European countries’ hospitals (1.37 per individual patient) as reported by Plachouras *et al*.^19^

There was little change in the number of orally and parenterally prescribed antimicrobials before and during the COVID-19 pandemic. Although consumption of parenteral antimicrobials was higher than oral therapy over the time, the percentage of parenteral therapy decreased across all timepoints, from 63.3% in 2009 to 56.2% in 2021, and so oral therapy increased from 37.7% in 2009 to 43.8% in 2021 within both hospital sites. This reduction of prescribed parenteral therapy could be explained in relation to an improvement in the prescribing pattern because parenteral therapy is usually associated with potential risks besides higher costs in treatment and hospitalizations than oral therapy.^20,21^

As per guidelines, patients with parenteral antimicrobial therapy should be reviewed within 72 h of starting the therapy to determine the appropriateness of switching to oral therapy.^22^ A stop or review date was recorded for 44.2% of patients on parenteral antimicrobial therapy during the pandemic, which was significantly higher than that recorded in the pre-pandemic year (8.7%). This significant reduction in parenteral antimicrobial prescriptions might have been potentially associated with a decrease in some risks and costs in the study sites; however, this might require further investigation. Schuts *et al*.^23^ showed that mortality risk decreased by 56% when modulating the parenteral antimicrobials into oral antimicrobials.

CAIs resulted in more antimicrobial prescriptions (68.6%) in the 2021 PPS data (during the pandemic) compared with 59.7% in the 2019 PPS data, whereas 16% of the antimicrobial prescriptions in the 2021 PPS data were attributed to HAIs, nearly half of that seen in the 2019 PPS data (31%). Treatment of respiratory infections accounted for the majority of the antimicrobial prescriptions over all timepoints; however, it was noted that the overall antimicrobial prescriptions for respiratory infections recorded during the pandemic (2021 PPS) was the lowest (31.3%) when compared with the pre-pandemic years across the six timepoints. This might be related to decreased patient visits for respiratory consultations and possibly the reduction of the infections due to implementation of public health measures such as less mixing of the population and social distancing restrictions.^24–26^

The major diagnosis in the 2021 PPS data was pneumonia or LRTIs, which was lower than that before the pandemic. The analysis showed that the 2021 PPS data during the pandemic were relatively consistent with those reported before in the results of PPS in 2017 in some European countries (23.2%), as reported by Vandael *et al.*^27^

Over time, the most frequently prescribed antimicrobial group was a combination of penicillins, including β-lactamase inhibitors (J01CR), which remained nearly constant before and during the pandemic (24% and 24.4%, respectively). Consumption of penicillins with extended spectrum (J01CA) reduced in the 2021 PPS (13.4%) compared with 17.8% in the 2019 PPS but was nearly consistent with the 13.7% in the 2015 PPS. The consumption of penicillins with extended spectrum (J01CA) raised over the timepoints and was found to be statistically significant between the six timepoints.

The major antimicrobial agents prescribed in the 2021 PPS (during the pandemic) were piperacillin/tazobactam, co-amoxiclav and ceftriaxone, which were similar to the 2019 PPS (before the pandemic). These findings were similar to those found in the EU PPS results published in 2016–2017.^19^ Broad-spectrum antimicrobial consumption (cephalosporins, co-amoxiclav, quinolones and clindamycin) represented 16% of all antimicrobial usage in the 2021 PPS, with all prescribing considered appropriate, which was lower than that in 2019 (19.4%). But this was contradicted by another study.^28^

Details of indications, therapy duration and date for review should be recorded against all antimicrobial prescriptions to help prescribers regularly make decisions about the antimicrobial therapy.^22^ Recording of indication improved over the timepoints, and the 2021 PPS value (95%) was consistent with that before the pandemic in the 2019 PPS (95.5%). There was an improved incidence of the recording of a date to review or stop antimicrobial therapy in the 2021 PPS (45.4%) compared with the 2019 PPS (20.9%). It is recommended that the continuity of prescribed antimicrobials should be checked and reviewed during the 72 h after starting the therapy.^29^

Antimicrobial guidelines-based resistance and severity patterns are considered to be the standard for antimicrobial stewardship programmes in addition to the cost and efficacy.^30,31^ The 2021 PPS results showed 66.2% of antimicrobial prescriptions were compliant with the local antimicrobial guidelines, slightly lower than the 69.5% in the 2019 PPS before the pandemic. The percentage of MP prescriptions increased over time, from 4.5% in 2009 to 8.3% in 2019 and 12% in 2021 (during the pandemic). The majority of the MP prescriptions in the 2021 PPS (89.6%) were non-assessable (no local guidelines for the indication). This was higher than the 2019 PPS (78.1%) before the pandemic. This finding might have resulted from concern regarding secondary bacterial infections during the fast and dynamic environment of the COVID-19 pandemic.^18^

Procalcitonin was used to reflect disease severity in the COVID-19 patients.^32^ However, biomarkers such as CRP and WBCs have been used frequently by physicians during the COVID-19 pandemic in order to differentiate between bacterial and viral infections and to assess the severity of COVID-19.^33–35^ The 2021 PPS showed that 81.5% of patients were prescribed antimicrobials based on biomarker data, which was slightly lower than the 83.5% in the 2019 PPS before the pandemic (but significantly higher than the 2015 finding of 61.5%), which reflects better compliance with the implemented antimicrobial stewardship programmes. CRP was the major biomarker used both pre-pandemic and during the pandemic for antimicrobial prescription decisions, directing 60.9% and 81% of patients’ therapy, respectively. Antimicrobial therapy should be directed by biomarker data to avoid an unnecessarily long therapy duration and hence costs.^36,37^

The 2021 PPS (during the pandemic) showed that 82.8% of antimicrobials were being prescribed empirically, comparable to a previous study.^38^ This proportion was less than before the pandemic in the 2019 PPS (89.7%). Antimicrobial consumption based on microbiology culture results leads to reduced costs and improved efficacy by reducing the therapy duration.^39^ Due to the high-paced environment and uncertainty during the COVID-19 pandemic, the 2021 PPS results showed that 54.4% of patients treated with an antimicrobial had a culture sent to the laboratory, which was lower than the 70.2% in the 2019 PPS before the pandemic.^40–42^ Microbiological culture data can direct clinicians towards the most appropriate antimicrobial therapy.^34,43^ Although only 17.2% of antimicrobial prescriptions were initiated by microbiological results in the 2021 PPS, this finding was an improvement on the 10.3% in the pre-pandemic 2019 PPS.

### Study limitations

This prevalence study was not suitable for measuring resistance rates; it explored the current situation within both hospitals. The PPS surveys do not collect data on resistance rates, which could be surveyed at a future timepoint to determine if they were impacted by the pandemic and associated prescribing changes.

The main data were collected from the biggest district hospitals within the Trust (Antrim and Causeway hospitals). However, some data from 2011 were collected from Craigavon Hospital (a different hospital from the two main hospitals included in the study) but are still comparable with the main data collected in 2019 and 2021.

In addition, there were differences in the protocols of the ECDC-PPS and the G-PPS, such as antivirals (J05) and antimalarials (P01B), which were only included in the G-PPS. Also, limited data about surgical prophylaxis prescriptions within the 2019 PPS before the pandemic made it hard to compare with the 2021 PPS during the pandemic and the other timepoints.

### Conclusions

There appears to be no impact of the COVID-19 pandemic on the overall antimicrobial prescribing patterns in two district hospitals in Northern Ireland. This observation may be attributed to the dedicated efforts in implementing and complying with antimicrobial stewardship programmes within both hospitals.

It was interesting to find some improvements in the 2021 PPS (during the pandemic) relative to before the pandemic in the 2019 PPS: a decreased number of antimicrobials prescribed per patient; a slight decrease in parenteral therapy and thus slight increase in oral therapy; the number of HAIs decreased to almost half its value in 2021 (during the pandemic) compared to 2019; an unexpected decrease in the incidence of respiratory infections; and a higher rate of stop or review date documented. It was found that the practice of recording a review or stop date for antimicrobials (alongside other factors such as enabling earlier discharges through reducing the length of parenteral therapy and HAIs) contributed to a slight improvemnet of overall 2021 PPS (during the pandemic) when compared to the pre-pandemic 2019 PPS. The antimicrobial stewardship programmes and guidelines played an important role in controlling antimicrobial consumption during the COVID-19 pandemic in the NHSCT, Northern Ireland.

Further studies are recommended to investigate the antimicrobial prescribing and resistance patterns with the inclusion of post-COVID-19 data.

## Supplementary Material

dlad117_Supplementary_Data

## Data Availability

Some of data contained in the study were recorded in the G-PPS database, which is accessible to healthcare professionals but with limited access to the public (https://www.global-pps.com/).

## Supplementary data

Table S1 is available as Supplementary data at *JAC-AMR* Online.
