# Supplementary material for: Antimicrobial prescribing in a secondary care setting during the COVID-19 pandemic
Source: JAC Antimicrob Resist. 2023 Nov 13;5(6):dlad117. doi: 10.1093/jacamr/dlad117 (PMC10642613; doi:10.1093/jacamr/dlad117)
Supplement: Supplementary file 1 [file dlad117_supplementary_data.docx]

  

   **No** 

**Ward Form (Fill in one form for each ward included in the PPS)** 

**Include only inpatients “admitted before and present at 08:00 hours” on the day of the PPS!** 

**Date of survey** (dd/mm/year) 

_____/_____/________ 

**Person completing form** (Auditor code) : 

**Hospital name :** 

**Ward Name :** 

**Adult wards** 

**Paediatric wards** 

**Ward Type:** 

 **AMW** (General or mixed **Adult Medical Ward**) 

 **ASW** (General or mixed **Adult Surgical Ward**) 

 **PMW** (Paediatric Medical Ward) 

Tick the most appropriate 

 **HO-AMW** (Haematology-Oncology) 

 **DIG-ASW** (Digestive tract surgery) 

 **HO-PMW** (Haematology-Oncology) 

type of department/ward 

 **T-AMW** (Transplant (BMT/solid)) 

 **ORT-ASW** (Orthopaedics-Trauma surg.) 

 **T-PMW** (Transplant (BMT/Solid)) 

 **P-AMW** (Pneumology) 

 **URO-ASW** (Urological surg.) 

 **PSW** (Paediatric Surgical Ward) 

 **CAR-AMW** (Cardiology) 

 **CV-ASW** (Cardio-vascular surg.) 

 **PICU** (Paediatric Intensive Care Unit)  

 **NEU-AMW**(Neurology) 

 **NEU-ASW** (Neurosurgery) 

 **ID-PMW** (Infectious Disease PMW) 

 **REN-AMW** (Nephrology) 

 **ONCO-ASW** (Oncology-cancer surg.) 

 **ID-AMW** (Infectious Disease) 

 **DB-AMW** (Dermatology-burn wards) 

 **PSY-AMW** (Psychiatry) 

 **REH-AMW** (Rehabilitation)  

 **GER-AMW** (Geriatrics) 

 **LTC-AMW** (Long-Term care) 

 **OBG-AMW** (gynaecology-obstetrics) 

 **PLAS-ASW** (Plastic, reconstructive surg.) 

 **ENT-ASW** (Ear-nose-throat surg.) 

 **AICU** (General or mixed **Adult Intensive Care Unit**) 

 **MED-AICU** (Medical AICU) 

 **SUR-AICU** (Surgical AICU) 

 **CAR-AICU** (Cardiac AICU) 

**Neonatal wards:**  

 **NMW** (Neonatal Medical Ward) 

 **NICU** (Neonatal Intensive Care Unit) 

**Mixed Ward** 

   **Yes**       

**Activity:** Tick as appropriate. In case of mixed wards, tick all encountered activities/specialities 

   **Medicine** 

   **Surgery** 

   **Intensive Care** 

**Total number of admitted inpatients** (=all patients whether they receive an antimicrobial or not !) 

on the ward present at 8.00 am on day of PPS. For mixed departments, fill the total number of 

patients corresponding to each of the encountered activities. 

**Total number of beds** on the ward present at 8:00 am on day of PPS split up by activity. For mixed 

departments fill in the total number of beds corresponding to each of the encountered activities. 

                                                                  **Global-PPS Data Collection Forms – May 2020** **(Basic PPS) - p1** 

fermenter Gram-negative bacilli 

22 

***Resistance type*** 

**to start antimicrobial** 

 25 

)**; 3GCREB** (3 

******** 

26 

; **Azoles** 

27 

1 

2 

3 

4 

5 

**6** 

7 

8 

9 

 10 

(see appendix II) 

(see appendix III) 

15 

16 

****** 

****** 

****** 

****** 

****** 

17 

18 

19 

20 

21 

 generation cephalosporin resistant 

23 

24 

.  Encode Microorganism also if resistance type is unknown. 

**GLOBAL-PPS PATIENT Form (Mandatory: Fill in one form per patient with an ongoing antimicrobial at 8am on the day of the PPS)** 

**Patient Age**   

***Current*** 

 ***Neonate only*** *(optional)* 

**Ward** **Name/code** 

**Activity**   

(M, S, IC) 

**Patient Identifier**   

**Survey Number**   

**Years** 

(if ≥ 2 

years) 

**Months** 

(1-23 

month) 

**Days** 

(if <1 

month) 

***Weight**** 

*In kg* 

***Gestatio-*** 

***nal age**** 

***Birth*** 

***weight* (kg)*** 

**Sex** 

M, F, U 

**Treatment based on biomarker data or WBC** 

0  Yes    

– 

0  No 

**Culture(s) sent to the lab to document infection* (Tick if yes)** 

**If yes, which:** 

CRP, PCT, other  

or WBC  

**Type** 

**biological** 

**fluid sample** 

(Blood/urine/ 

other) 

**Most relevant value close** 

        **Value          Unit**  

 

 

**Blood**   

**Urine**  

  **Cerebrospinal fluid** 

  **Wound (surgery/biopsy)** 

  **BAL (protected resp. specimen)** 

  **Sputum/bronchial aspirate** 

  **Other type of specimen** 

**Antimicrobial Name**   

**1.** 

**2.**  

**3.**  

**4.**  

**5.** 

**Start date of the antimicrobial* (*****dd/mm/yyyy)*** 

**Single Unit Dose**   

**Unit** (g, mg, IU, MU)   

**Doses/ day** 

**Route**  (P, O, R, I) 

11 

**Diagnosis** 

12 

**Type of indication** 

13 

**Reason in Notes** (Yes or No) 

14 

**Guideline Compliance** (Y, N, NA, NI) 

**Is a stop/review date documented?**(Yes/No) 

**Treatment**  (E: Empirical; T: Targeted) 

**The following resistance data is to be filled in only if the treatment choice is based on microbiology data (Treatment=T) available on the day of the PPS** 

**Maximum 3 microorganisms (MO) to report** 

**Maximum 1 Resistance type by MO to report** 

**MO** 

**R type** 

**MO** 

**R type** 

**MO** 

**R type** 

**MO** 

**R type** 

**MO** 

**R type** 

**Insert codes (see Appendix IV, page 9)          MO 1**  

**MO 2** 

**MO 3** 

- choose between: **MRSA**  ;  **MRCoNS  ;  PNSP  ; MLS  ; VRE**  ; **ESBL** (ESBL-producing Enterobacterales 

rd 

Enterobacterales);  **CRE** (Carbapenem-resistant Enterobacterales   )**;  ESBL-NF** (ESBL-producing non fermenter Gram-negative bacilli  );  **CR-NF** (Carbapenem-resistant non 

);  **other MDRO** 

**Note: *** ***Current weight***, **Gestational age (in number of weeks),** ***Birth weight***, ***Start date of the antimicrobial*** and ***Cultures sent to the lab*** are **optional variables**.  

                                                                  **Global-PPS Data Collection Forms – May 2020** **(Basic PPS) - p2** 

27 

26   

25  

23 

22 

21 

20 

19 

18 

17 

16  

15 

14 

13 

12 

11 

10 

9 

8 

7 

6    

4 

3 

2 

1 

: 

   Activity: M=medicine (including Psychiatric cases, *etc.*), S=surgery (including orthopaedics, obstetrics and gynaecology, *etc.*), IC=intensive care 

   Patient Identifier: A unique patient identifier that allows linkage to patient records at local level for more detailed audit. This unique identifier will not be included in the 

online database. 

   Survey Number: A unique non-identifiable number given by WebPPS for each patient entered in the database.  Leave blank but note down the number after the patient 

data has been recorded in the online database. The number is displayed once (and only) after the patient data has been recorded in the online database.  

   Patient Age: If the patient is 2 years old or older, specify only the number of years, if between 1 and 23 months specify only the number of months, if less than 1 month  

specify the number of days. 

5 

   If treatment based on biomarker, specify which one: **CRP** (C-reactive protein), **PCT** (Procalcitonin), **Other** lab-based biomarker other than CRP, PCT; or **WBC** (white blood 

cell count). 

The unit for the biomarker CRP or PCT value expressed in mg/L, μg/L, ng/L, mg/dL, ng/dL, ng/mL, μg/mL, nmol/L.  

In thousand per microliter (μL) for WBC count (normal  number of WBCs in the blood is 4,500 to 11,000 WBCs per microliter). 

For a conversion calculator see:[ ](http://unitslab.com/node/67)[h](http://unitslab.com/node/67)[tt](http://unitslab.com/node/67)[p](http://unitslab.com/node/67)[:](http://unitslab.com/node/67)[/](http://unitslab.com/node/67)[/](http://unitslab.com/node/67)[u](http://unitslab.com/node/67)[n](http://unitslab.com/node/67)[itslab](http://unitslab.com/node/67)[.co](http://unitslab.com/node/67)[m](http://unitslab.com/node/67)[/](http://unitslab.com/node/67)[n](http://unitslab.com/node/67)[o](http://unitslab.com/node/67)[d](http://unitslab.com/node/67)[e/](http://unitslab.com/node/67)[6](http://unitslab.com/node/67)[7](http://unitslab.com/node/67)[ ](http://unitslab.com/node/67)[(CRP) and](http://unitslab.com/node/67)[ ](http://unitslab.com/node/103)[h](http://unitslab.com/node/103)[tt](http://unitslab.com/node/103)[p](http://unitslab.com/node/103)[:](http://unitslab.com/node/103)[/](http://unitslab.com/node/103)[/](http://unitslab.com/node/103)[u](http://unitslab.com/node/103)[n](http://unitslab.com/node/103)[itslab](http://unitslab.com/node/103)[.co](http://unitslab.com/node/103)[m](http://unitslab.com/node/103)[/](http://unitslab.com/node/103)[n](http://unitslab.com/node/103)[o](http://unitslab.com/node/103)[d](http://unitslab.com/node/103)[e](http://unitslab.com/node/103)[/](http://unitslab.com/node/103)[1](http://unitslab.com/node/103)[0](http://unitslab.com/node/103)[3](http://unitslab.com/node/103)[ ](http://unitslab.com/node/103)[(procalcitonin).](http://unitslab.com/node/67) 

   Antimicrobial Name: Insert generic name. 

   Single Unit Dose: Numeric value for dose per administration (in grams, milligrams, IU or MU). 

  Unit: The unit for the dose (g, mg, IU or MU)   

  Doses/day If necessary provide fractions of doses: (e.g., every 16h = 1.5 doses per day, every 36h = 0.67 doses per day, every 48h = 0.5 doses per day) 

   Route: Routes of administration are: Parenteral (P), Oral (O), Rectal (R), Inhalation (I). 

  See diagnoses groups list (Appendix II) 

  See Indication codes (Appendix III) 

  Reason in Notes**:** A diagnosis / indication for treatment is recorded in the patient’s documentation (treatment chart, notes, etc.) at the start of antibiotic course (Yes or 

No) 

 Guideline Compliance: Refers to antibiotic choice (not route, dose, duration  etc) in compliance with **local** guidelines (Y: Yes; N: No; NA: Not Assessable because of 

absence of local guidelines for the specific indication;  NI: No Information because diagnosis/indication is unknown) 

Treatment: **Report “E”** 1) when the antibiotic is being used as per a local guideline, treatment by which experience has proved to be beneficial; 2) when a culture or 

microbiological examination is not done ; 3) when a microbiological examination is done, BUT not yet available on the day of the PPS; or the result was not assessable. 

**Report “T”** if based upon microbiological result; Report also “T” if the micro-organism yielded susceptible results.   

  Methicillin-resistant *Staphylococcus aureus* (MRSA) 

 Methicillin-resistant coagulase negative staphylococci (MRCoNS)  

  Penicillin-non susceptible *Streptococcus pneumoniae* (PNSP) 

  Macrolide-lincosamide-streptogramin resistance in Streptococcus isolates (MLS) 

 Vancomycin-resistant enterococci (VRE) 

  Bacteria, producing extended-spectrum beta-lactamases (ESBL)  

  Carbapenem-resistant *Enterobacterales* (CRE) – enteric bacteria resistant to imipenem, meropenem or other carbapenems 

24   

ESBL Non fermenters (ESBL-NF): *Pseudomonas aeruginosa, Acinetobacter baumannii, Burkholderia spp., Stenotrophomonas maltophilia* multidrug resistant 

Carbapenem-resistant  Nonfermenters (CR-NF) – nonfermenters  resistant to imipenem, meropenem or other carbapenems 

Multi-drug resistant (MDR) pathogens, others than the listed above 

  Azoles: if the medicinal product chosen is intended to treat infections caused by azole-resistant fungi and yeasts (e.g*. Candida spp*., *Aspergillus spp*.) 

                                                                  **Global-PPS Data Collection Forms – May 2020** **(Basic PPS) - p3** 

**Appendix I: Combination anti-infective agents** 

Combinations of an antibiotic and a beta-lactamase inhibitor: 

Ampicillin and beta-lactamase inhibitor: report only ampicillin dose (J01CR01) 

Amoxicillin and beta-lactamase inhibitor: report only amoxicillin dose (J01CR02) 

Ticarcillin and beta-lactamase inhibitor: report only ticarcillin dose (J01CR03) 

Piperacillin and beta-lactamase inhibitor: report only piperacillin dose (J01CR05) 

Imipenem and beta-lactamase inhibitor: report only imipenem dose (J01DH51) 

Panipenem and betamipron: report only panipenem (J01DH55) 

Example: 

Amoxicillin and beta-lactamase inhibitor 1.2g IV 🡪 1g (amoxicillin) + 200mg (clavulanic acid), **report** 

**only 1 g as a dose** 

Piperacillin and beta-lactamase inhibitor 4.5g IV 🡪 4g (piperacillin) + 500mg (tazobactam), **report** 

**only 4 g as a dose** 

Other combinations of multiple antimicrobial substances: 

J01EE01 Sulfamethoxazole and Trimethoprim: **report the total amount of** 

**sulfamethoxazole and trimethoprim** 

Example: 

Co-trimoxazole 960mg: (sulfamethoxazole. 800mg + trimethoprim 160mg), **report 960mg** 

Further information on agents included for the Global-PPS is available in the antimicrobial list. Only 

antimicrobial substance name need to be written down, NOT the ATC codes!  (excel file - available at 

website under documents: Global-PPS_antimicrobial_list.xlsx)[ ](http://www.global-pps.com/)[h](http://www.global-pps.com/)[tt](http://www.global-pps.com/)[p](http://www.global-pps.com/)[:](http://www.global-pps.com/)[//](http://www.global-pps.com/)[ww](http://www.global-pps.com/)[w.g](http://www.global-pps.com/)[lob](http://www.global-pps.com/)[a](http://www.global-pps.com/)[l](http://www.global-pps.com/)[-](http://www.global-pps.com/)[p](http://www.global-pps.com/)[p](http://www.global-pps.com/)[s.co](http://www.global-pps.com/)[m](http://www.global-pps.com/)[/](http://www.global-pps.com/)[ ](http://www.global-pps.com/)

                  **Global-PPS Data Collection Forms – May 2020 (Basic PPS) - p4** 

**Appendix II - Diagnostic codes (what the clinician aims at treating)**  

**Site** 

**Codes** 

**Examples** 

**CNS** 

**Proph CNS** 

Prophylaxis for CNS (neurosurgery, meningococcal) 

**CNS** 

Infections of the **C**entral **N**ervous **S**ystem 

**EYE** 

**Proph EYE** 

Prophylaxis for Eye operations 

**EYE** 

Therapy for Eye infections e.g., Endophthalmitis 

**ENT** 

**Proph ENT** 

Prophylaxis for **E**ar, **N**ose, **T**hroat (**Surgical or Medical prophylaxis=SP/MP**) 

**ENT** 

Therapy for **E**ar, **N**ose, **T**hroat infections including mouth, sinuses, larynx 

**AOM** 

Acute otitis media 

**RESP** 

**Proph RESP** 

Pulmonary surgery, prophylaxis for **Resp**iratory pathogens e.g. for aspergillosis  

**LUNG** 

Lung abscess including aspergilloma 

**URTI** 

**U**pper **R**espiratory **T**ract viral **I**nfections including influenza but not ENT 

**Bron** 

Acute **Bron**chitis or exacerbations of chronic bronchitis 

**Pneu** 

**Pneu**monia or LRTI (lower respiratory tract infections) 

**COVID-19** 

Coronavirus disease caused by SARS-CoV-2 infection 

**TB** 

Pulmonary TB (Tuberculosis) 

**CF** 

Cystic fibrosis 

**CVS** 

**Proph CVS** 

**C**ardiac or **V**ascular Surgery, endocarditis prophylaxis 

**CVS** 

**C**ardio**V**ascular **S**ystem infections: endocarditis, endovascular device e.g pacemaker, vascular graft 

**GI** 

**Proph GI** 

**G**astro-**I**ntestinal tract surgery, liver/biliary tree, GI prophylaxis in neutropenic patients or hepatic failure 

**GI** 

Gastro-Intestinal infections (salmonellosis, *Campylobacter*, parasitic, etc.) 

**IA** 

**I**ntra-**A**bdominal sepsis including hepatobiliary, intra-abdominal abscess *etc*. 

**CDIF** 

*Clostridioides difficile* infection 

**SSTBJ** 

**Proph BJ** 

Prophylaxis for SST, for plastic or orthopaedic surgery (**B**one or **J**oint)  

**SST** 

**S**kin and **S**oft Tissue: Cellulitis, wound including surgical site infection, deep soft tissue not involving 

bone e.g., infected pressure or diabetic ulcer, abscess 

**BJ** 

**B**one/**J**oint Infections: Septic arthritis (including prosthetic joint), osteomyelitis 

**UTI** 

**Proph UTI** 

Prophylaxis for urological surgery **(SP)** or recurrent **U**rinary **T**ract **I**nfection **(MP)** 

**Cys** 

Lower Urinary Tract Infection (UTI) : cystitis 

**Pye** 

Upper UTI including catheter related urinary tract infection, pyelonephritis  

**ASB** 

Asymptomatic bacteriuria 

**GUOB** 

**Proph OBGY** 

Prophylaxis for **OB**stetric or **GY**naecological surgery (SP: section caesarean, no episiotomy; MP: 

carriage of group B streptococcus) 

**OBGY** 

**Ob**stetric/**Gy**naecological infections, **S**exually **T**ransmitted **D**iseases (**STD**) in women 

**GUM** 

**G**enito-**U**rinary **M**ales + Prostatitis, epididymo-orchitis, STD in men 

**No** 

**BAC** 

Bacteraemia or fungaemia with no clear anatomic site and no shock 

**defined** 

**SEPSIS** 

Sepsis of any origin (eg urosepsis, pulmonary sepsis etc), sepsis syndrome or septic shock with no 

**site** 

clear anatomic site. Include fungaemia (candidemia) with septic symptoms 

**(NDS)** 

**Malaria** 

**HIV** 

Human immunodeficiency virus 

**PUO** 

**P**yrexia of **U**nknown **O**rigin - Fever syndrome with no identified source or site of infection  

**PUO-HO** 

Fever syndrome in the non-neutropenic **H**aemato–**O**nco patient with no identified source of pathogen  

**FN** 

**F**ever in the **N**eutropenic patient  

**LYMPH** 

**Lymph**atics as the primary source of infection eg suppurative lymphadenitis 

**Sys-DI** 

Disseminated infection (viral infections such as measles, CMV …)  

**Other** 

Antimicrobial prescribed with documentation but no defined diagnosis group 

**MP-GEN** 

Drug is used as **M**edical **P**rophylaxis in **gen**eral, without targeting a specific site, e.g. antifungal 

prophylaxis during immunosuppression 

**UNK** 

Completely **Unk**nown Indication 

**PROK** 

Antimicrobial (e.g. erythromycin) prescribed for **Prok**inetic use 

**Neo-** 

**MP-MAT** 

**M**edical **P**rophylaxis for **Maternal** risk factors e.g. maternal prolonged rupture membranes 

**natal** 

**NEO-MP** 

Drug is used as **M**edical **P**rophylaxis for **Newborn** risk factors e.g. VLBW (Very Low Birth Weight) and 

IUGR (Intrauterine Growth Restriction) 

**CLD** 

Chronic lung disease: long-term respiratory problems in premature babies (bronchopulmonary 

dysplasia) 

                  **Global-PPS Data Collection Forms – May 2020 (Basic PPS) - p5** 

**Inter-** 

**vention** 

**related** 

**HAI** 

**APPENDIX III - Type of Indication** 

**CAI**  Community 

Symptoms started ≤ 48 hours from admission to hospital (or present on 

acquired infection 

admission). 

**HAI1**  Post-operative surgical site infection (within: 30 days of surgery OR;       

**HAI**   

90 days after implant surgery) 

Healthcare    

Associated        

Infection: 

Symptoms  

**HAI2**  ***Intervention*** related infections of mixed origin (mix of CVC-BSI, PVC-BSI, 

VAP, CAUTI; or related to tubes/drains) 

**HAI2-CVC-BSI**  (Central Venous **Catheter**-related Blood Stream Infection) 

start           

**48 hours**  

**HAI2-PVC-BSI**  (Peripheral Vascular **Catheter**-related Blood Stream Infection) 

**after**  

**HAI2-VAP**  (Ventilator Associated **Pneumonia**) 

**admission**                  

to hospital  

**HAI2- CAUTI**  (Catheter Associated **Urinary** Tract Infection) 

**HAI3**  *C. difficile* associated diarrhoea (CDAD) (>48 h post-admission or <30 

days after discharge from previous admission episode. 

**HAI4**  Other hospital acquired infection of mixed or undefined origin (HAP, UTI, 

BSI) 

**HAI4-BSI Blood** Stream Infection, not intervention related 

**HAI4-HAP**  Non-intervention related Hospital Acquired **Pneumonia** (not VAP) 

**HAI4-UTI  Urinary** Tract Infection, not intervention related 

**HAI5**  Patient readmitted <48h after stay in another hospital, with infection 

present on current admission or within 48 hours (patient with infection from 

another hospital) 

**HAI6**  Infection present on admission from long-term care facility (LTCF) or 

Nursing Home***** 

**SP**  Surgical 

prophylaxis****** 

**SP1** Single dose 

**SP2** one day 

**SP3**  >1 day 

For **surgical patients**, administration of prophylactic antimicrobials **should be checked in the previous** 

**24 hours** in order to encode the duration of prophylaxis as either one dose, one day (= multiple doses 

given within 24 hours) or >1 day.  

See more explanation and **table** in **protocol page 8** !  

**MP**  Medical 

For example long term use to prevent UTI’s or use of antifungals in patients 

prophylaxis 

undergoing chemotherapy or penicillin in asplenic patients *etc*.  

**OTH**  Other 

For example erythromycin as a motility agent (motilin agonist). 

**UNK** 

Completely unknown indication 

**Select 1 possibility for each reported antimicrobial** 

*Long-term care facilities represent a heterogeneous group of healthcare facilities, with care ranging from 

social to medical care. These are places of collective living where care and accommodation is provided as 

a package by a public-agency, non-profit or private company (e.g. nursing homes, residential homes). 

**Surgical prophylaxis includes those antibiotics prescribed before and after a surgical intervention 

(surgery in the operation room). The code SP1, SP2, SP3 goes with a diagnostic code preceded by 

‘proph’ (e.g. ‘proph GI’) 

                  **Global-PPS Data Collection Forms – May 2020 (Basic PPS) - p6** 

**APPENDIX IV – list of micro-organisms by resistance type** 

                  **Global-PPS Data Collection Forms – May 2020 (Basic PPS) - p7**
